# Biological mechanisms of gold nanoparticle radiosensitization

**DOI:** 10.1186/s12645-017-0026-0

**Published:** 2017-02-02

**Authors:** Soraia Rosa, Chris Connolly, Giuseppe Schettino, Karl T. Butterworth, Kevin M. Prise

**Affiliations:** 1 Centre for Cancer Research and Cell Biology, Queens University Belfast, 97 Lisburn Road, Belfast, BT9 7AE Northern Ireland, UK; 2 National Physical Laboratory, Teddington, London, TW11 0LW UK

**Keywords:** Cancer therapy, Radiation therapy, Radiosensitization, Gold nanoparticle

## Abstract

There has been growing interest in the use of nanomaterials for a range of biomedical applications over the last number of years. In particular, gold nanoparticles (GNPs) possess a number of unique properties that make them ideal candidates as radiosensitizers on the basis of their strong photoelectric absorption coefficient and ease of synthesis. However, despite promising preclinical evidence in vitro supported by a limited amount of in vivo experiments, along with advances in mechanistic understanding, GNPs have not yet translated into the clinic. This may be due to disparity between predicted levels of radiosensitization based on physical action, observed biological response and an incomplete mechanistic understanding, alongside current experimental limitations. This paper provides a review of the current state of the field, highlighting the potential underlying biological mechanisms in GNP radiosensitization and examining the barriers to clinical translation.

## Background

**Fig. 1:**
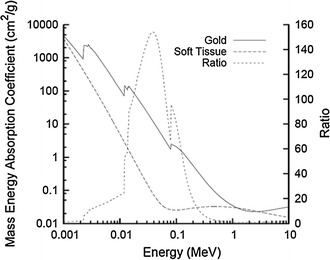
Photon mass energy absorption coefficients of soft tissue and gold. The ratio of the mass energy absorption coefficients is shown as a function of photon energy (Hubbell and Seltzer 1996)

Radiation therapy is frequently used in the treatment of cancer, with both curative and palliative intent. However, radiation doses that can be delivered to patients are limited by toxicity in the surrounding healthy tissue. Many efforts in Radiation Oncology have focussed on approaches that aim to preferentially sensitize tumours to radiation whilst minimizing effects in normal tissues. An approach to maximize the differential response between tumour and normal tissue response, termed therapeutic ratio, is through the introduction of high-atomic number (*Z*) material into the target. Gold (*Z* = 79) is a promising radiosensitizer in this regard due to its high atomic number and mass energy coefficient relative to soft tissue. As shown in Fig. 1, the mass energy coefficient of gold is 100–150 times greater than that of soft tissue in the keV energy range (Hubbell and Seltzer 1996). Consequently, there is an increased probability of photoelectric interaction at lower energy levels, resulting in increased energy deposition at the target site. However, considering the depth dose limitations of keV X-rays, MV energies are used as the clinical standard for external beam radiotherapy. At these energies, significant radiosensitization would not be expected based on the ratio of mass energy absorption coefficients of gold and soft tissue.

**Fig. 2:**
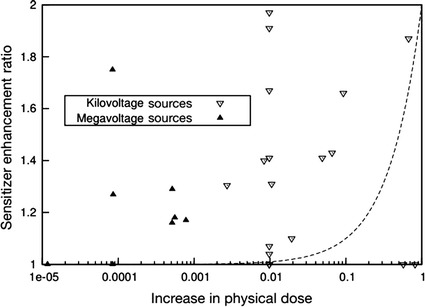
Comparison of predicted and observed values of dose enhancement for gold nanoparticles at both megavoltage and kilovoltage energies. “Increase in physical dose” here refers to the ratio of the additional dose deposited by X-rays in the system due to the addition of GNPs to that which would be deposited in the absence of gold. The observed data in this figure are dose modification results from in vitro experiments, while the predicted dose increase is based on the gold concentrations and X-ray energies used. The *dashed line* shows the trend which would be followed if the sensitizer enhancement ratio directly followed predicted increases in physical dose (Butterworth et al. 2013)

Despite this, observed experimental findings deviate from predicted levels of radiosensitization, with effects observed at concentrations lower than predicted. Figure 2 shows the X-ray dose modification results against the predicted degree of dose enhancement, based on the gold concentrations and X-ray energies used for a range of experimental studies (Butterworth et al. 2012). The observed degree of radiosensitization in almost all of the experimental results is much greater than the predicted increase in physical dose. Physical dose variation between differing nanoparticle preparations and cell lines can be seen to be much smaller than predicted, highlighting this as an unknown in cellular radiobiological response which is not driven by increasing the total dose delivered to the cells behind the radiosensitizing properties of gold nanoparticles (Butterworth et al. 2013).

The ISO international standard defines nanoparticles as ‘particles which typically do not exceed 100 nm in any of their dimensions’ (Michael et al. 2005). GNPs consist of a gold core which can be generated from various synthesis techniques to give rise to a wide range of different sizes and shapes, covered by a surface coating (Grzelczak et al. 2008). The surface coating can be functionalized for several uses, such as imaging, delivery and diagnostics (Rana et al. 2012; Ghosh et al. 2008; Mieszawska et al. 2013; Sperling et al. 2008).

This review follows on from our 2012 paper “Physical basis and biological mechanisms of gold nanoparticle radiosensitization” and aims to review the current state of the field. This will be accomplished by addressing three main points: the physical basis of GNP radiosensitization; the biological mechanisms of GNP radiosensitization; and the uptake, imaging potential and toxicity of GNPs in biological systems.

## Physical basis of GNP radiosensitization

It is known that ionizing radiation can directly or indirectly damage DNA and disrupt the atomic structure of other biomolecules (Kavanagh et al. 2013; Azzam et al. 2012). DNA repair mechanisms may fail leading cells to stop dividing, die or be mis-repaired, thus acquiring mutations that can result in malignant transformation (Begg et al. 2011). Therefore, avoiding normal tissue is of significant importance in reducing secondary side effects of radiotherapy.

However, one of the major challenges of radiotherapy is its lack of selectivity due to the similar mass energy absorption properties of both cancer and healthy tissues (Butterworth et al. 2012). In order to overcome this, agents such as metal-based nanoparticles (with high *Z*) have been found to improve the contrast between tumour and soft tissues, thus presenting radiosensitizing properties and potentially improving tumour control, reducing side effects and increasing survival when compared to radiotherapy alone (Herold et al. 2000; Regulla et al. 2002). Those absorb more energy per unit mass than soft tissue increasing the local dose deposited in the tumour (Hubbell and Seltzer 1996).

**Fig. 3:**
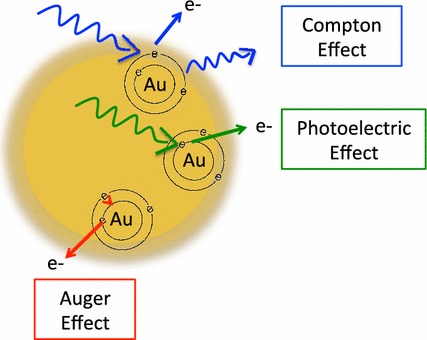
Schematic illustration of the photoelectric, Compton and Auger Effects. The Compton effect is represented in *blue*, the photoelectric effect in *green* and the Auger effect in *red* as described above

The main physical mechanisms through which radiation interacts with nanoparticles in the keV range are the Compton and Photoelectric effects, where an incident photon can either be partially or fully absorbed by an electron from the atom, causing its ejection (McMahon et al. 2016). The Photoelectric effect is a competing process in which the electrons are ejected preferentially from an inner atomic orbital. The vacancy left can then be filled by another outer shell electron that falls to its place, further releasing low-energy photons promoting a cascade release of secondary electrons (Butterworth et al. 2012). This process is called the Auger cascade and it is the major contribution to the production of low-energy electrons that have a range of few micrometres and cause highly localized ionizing events (Fig. 3) (Butterworth et al. 2012; Xie et al. 2015).

High-Z elements such as iodine, gadolinium and gold have been shown to have the ability to image and radiosensitize tumours (Herold et al. 2000; Regulla et al. 2002; Luchette et al. 2014; Martin 2002). Furthermore, gold has been shown to be biocompatible which makes it an ideal candidate as a radiosensitizer (Shukla et al. 2005; Hainfeld et al. 2010; Hainfeld et al. 2004).

GNPs were initially thought to enhance radiosensitization through physical processes but extensive data have shown chemical and biological components involved in the radiosensitization process as there is a poor correlation between experimental biological results and dosimetric calculations (Butterworth et al. 2012; Ionita et al. 2005; Jain et al. 2011).

Simulations suggest that the presence of 1% of gold could double the dose deposited using X-rays at keV energies and experimental evidence has demonstrated their ability to radiosensitize (Cho 2005). On the other hand, theoretical calculations predict that using MV X-ray sources there would be no significant increase in the dose deposited (Butterworth et al. 2012, 2013). Recently, it has been demonstrated by Monte Carlo simulations that there is an increase in secondary electron production when gold is irradiated with X-rays at 6 MV compared to water (Ka 2015). Also, in vitro and in vivo experimental evidence demonstrates a higher radiosensitization effect compared to the predicted increase in physical dose expected, in this energy range, suggesting a strong biological component in the radiosensitization process as shown in Fig. 2.

## Biological mechanisms of GNP radiosensitization

**Fig. 4:**
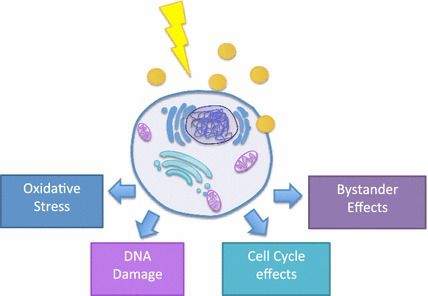
Schematic representation of the biological mechanisms involved in GNP radiosensitization. GNPs influence oxidative stress, DNA damage, cell cycle and bystander effects

The main mechanisms identified as being involved in the biological response of cells to gold nanoparticle radiosensitization are the production of ROS and oxidative stress, DNA damage induction, cell cycle effects and potential interference with the bystander effects (Fig. 4) as described in the following sections in more detail.

### ROS and oxidative stress

Gold is believed to be chemically inert. However, growing evidence suggests that their surface is electronically active, thus catalysing chemical reactions and promoting an increase in the production of ROS (Ionita et al. 2005; Mikami et al. 2012; Ionita et al. 2007; Zhang et al. 2003). This seems to be more evident in small NPs (nanoparticles) <5 nm in diameter that present a greater surface area-to-volume ratio (Li 2006; Hvolbæk et al. 2007; Cheng et al. 2012). One of the identified mechanisms as a possible reason for cytotoxicity is through the interaction of the NP surface with O_2_. In this process, donor electrons are transferred from the surface of the NPs to oxygen molecules generating superoxide, which can lead to ROS production through dismutation. This has been identified for single-component materials and for transition metals on the nanoparticle surface, such as Fe and vanadium, which take part in the formation of active sites (Li 2006).

In addition to reactive radicals on the NP surface, there are other sources of oxidative stress such as the redox groups in the coating, contaminants from the production method of non-metal NPs and oxidant-inducing properties of NPs (Li 2006; Fard et al. 2015; Tournebize et al. 2012). Oxidative stress causes damage to cell membranes, DNA and protein being identified so far as one of the major causes of NP cytotoxicity (Pan et al. 2009; Xia et al. 2006). Several reports of ROS production and oxidative stress induced by nanoparticles alone have been published as well as in combination with ionizing radiation as discussed below.

#### Nanoparticle-induced oxidative stress

In vitro reports have shown enhanced ROS production in the presence of GNPs and the absence of radiation by various groups (Li 2006; Pan et al. 2009; Coulter et al. 2012; Wahab et al. 2014; Chompoosor et al. 2010; Tang et al. 2015; Mateo et al. 2014). Mitochondria seem to play a role in it with data indicating loss of function due to high intracellular ROS levels. ROS can oxidize the mitochondrial membrane disrupting its potential and leaking more superoxide anions into the cytosol which can in turn be converted into H_2_O_2_ molecules. These further diffuse across membranes and damage DNA (Hei 2015; Havaki et al. 2015).

This is supported by experimental findings using 1.4-nm triphenyl monosulfonate (TPPMS)-coated GNPs that promote loss of mitochondrial potential through elevated oxidative stress causing necrotic cell death (Pan et al. 2009). Moreover, it was also found that antioxidants containing thiol groups bind to the surface of GNPs. This suggests that GNPs can bind these antioxidants inside cells inhibiting endogenous reducing agents from acting and therefore reduce the redox capacity of the cells (Pan et al. 2009). GNPs with different sizes, shapes and surface properties can also promote apoptosis or necrosis through ROS generation (Coulter et al. 2012; Wahab et al. 2014; Chompoosor et al. 2010; Tang et al. 2015; Mateo et al. 2014). Tiopronin-coated GNPs led to necrosis by enhanced ROS production after 24-h exposure in HeLa and L929 (fibroblast) cells (Li 2006). Also, dose dependency has been found to increase ROS production with citrate GNPs. These lead to apoptosis due to mitochondrial dysfunction with associated upregulation of caspase 3 and 7 (Wahab et al. 2014). Furthermore, mitochondrial membrane polarization is decreased and mitochondrial oxidation is increased when cells are exposed to GNPs (AuroVistTM) (Taggart et al. 2014). Moreover, in an indirect way, Au (gold) clusters (Au25peptide9) have been shown to dramatically increase ROS production via inhibition of thioredoxin reductase 1 (TrxR1) activity (Liu et al. 2014). TrxR1 is a regulator of redox reactions within cells and its binding to the GNP surface dramatically increased ROS levels inducing apoptosis (Arnér and Holmgren 2000; Fang et al. 2005; Tonissen and Trapani 2009; Omata et al. 2006).

In combination with ionizing radiation, GNPs contribute to an increased radiosensitization through which enhanced radical production has been observed following irradiation in the presence of glucose-capped GNPs with 90 kVp and 6 MV X-rays (Geng et al. 2011). Also, GNPs in water exposed to 100 kVp X-rays can promote an increase in hydroxyl radicals (1.46 fold) and superoxide anions (7.68-fold) (Misawa and Takahashi 2011). These increased levels of ROS and oxidative stress can trigger apoptosis as observed with 14-nm particles, in ovarian cancer cells, exposed to a MV and kV X-ray source (Geng et al. 2011).

Taggart et al. (2016) established a biological mechanism significantly contributing to radiosensitization, using 1.9-nm thiol-coated GNPs. Irradiation in the presence of GNPs led to an interaction between GNPs and the cell membrane protein disulfide isomerase (PDI), resulting in the disruption of thiol balance within the cell, thus causing cellular redox imbalance and ultimately oxidative stress. This leads to significant increases in cell killing, causing the GNPs to act as radiosensitizers. Variation in the expression levels of PDI in cancerous cells provides some insight into the range of radiosensitization observed across cell types.

In the next section, we highlight situations where radiosensitization enhancement has been achieved resorting to nanoparticles.

#### Nanoparticle-enhanced radiosensitization

Gold nanoparticle-mediated enhanced radiosensitization has been achieved by several research groups.

It was found using 50-nm GNPs that radiosensitized HeLa cells when irradiated with 220 kVp X-rays giving a DEF (dose enhancement factor) of 1.43 greater than that observed for smaller nanoparticles (from 14 to 74 nm). The cellular uptake rate was higher for the 50-nm GNPs, which was correlated with an increased radiosensitization, compared to the smaller GNPs tested, and concentration dependent (Chithrani et al. 2010). Also, functionalized Glu (thioglucose)-GNPs and AET (cysteamine)-GNPs exposed to 200 kVp X-rays and gamma rays demonstrated a significant increase in cell death in breast cancer cells compared to naked GNPs (Kong et al. 2008). Further experiments demonstrated that 1.9-nm GNPs (AuroVistTM) following 2 Gy radiation exposure (225 kVp) radiosensitize cells increasing apoptotic levels (Taggart et al. 2014). Moreover, in vitro studies have demonstrated GNPs’ ability to radiosensitize tumours at clinically relevant energies. At highest energies (6 MV), this has been shown by Chithrani et al. (2010). This is supported by 1.9-nm GNPs (AuroVistTM) that radiosensitize at 6 MV, and 15 MV X-rays with DEFs of 1.29 and 1.16 in MDA-MB-231 cells (Jain et al. 2011).

Hainfeld demonstrated evidence of radiosensitization in mammary tumour-bearing mice using 1.9-nm GNPs and 250 kVp X-ray radiation. Mice irradiated together with GNPs had 86% 1-year survival contrasting with 20% for X-rays alone (Hainfeld et al. 2004). Furthermore, citrate-coated GNPs that had a low radiosensitization effect in vitro with a DEF of 1.08 promoted a delay in tumour growth in B16F10 murine melanoma model. This was accompanied with increased survival from 20 days for non-irradiated mice to 55 days for mice irradiated with 6 MV and 65 days for mice irradiated with GNPs (Chang et al. 2008).

Although radiosensitization can be observed in some cell lines, others like DU145 human prostate cancer cells that uptake GNPs do not show significant effects at kV nor at MV energies (sensitization enhancement ratio: 0.97–1.08) (Jain et al. 2011). A summary of radiosensitizing experiments combining GNPs and ionizing radiation is presented in Table 1. Cumulatively, these data strongly suggest a significant biological component in GNP radiosensitization; however, the exact cellular mechanisms remain to be fully elucidated.

**Table 1:** Summary of radiosensitizing experimental data obtained with ionizing radiation and gold nanoparticles

Author	Size (nm)	Concentration	Surface coating	Cell model	Source energy	DEF/effect
In vitro						
Bobyk et al. (2013)	1.9 15	10 mg/ml, 15 min	Thiol	F98 glioma cells	50 keV, 6 Gy	Sensitization ratio of 1.92 for 1.9 nm particles and 1.4 for 15 nm particles
Butterworth et al. (2010)	1.9	10, 100 µg/ml	Thiol	AGO-1552B Astro DU-145 L132 MCF-7 MDA-MB-231 PC-3 T98G	160 kVp	1.97 0.96 0.81 0.87 1.09 1.11 1.02 1.91
Chang et al. (2008)	13	10 nM	Citrate	B16F10	6 MV e^−^	Significant decrease in SF @ 8 Gy
Chattopadhyay et al. (2013)	30	2.4 mg/ml	PEG, HER2 targeted	MDA-MB-231	100 kVp	1.6 (targeted) 1.3 (untargeted)
Chen et al. (2015)	28	36 µg/ml	BSA	U87	160 kVp	1.37
Chithrani et al. (2010)	14 50 74	7 × 10^9^ NPs/ml	Citrate	HeLa	105 kVp 220 kVp 660 keV 6 MV	1.66 1.43 1.18 1.17
Coulter et al. (2012)	1.9	12 µM (500 µg/ml)	Thiol	MDA-MB-231 DU-145 L132	160 kVp	1.8
Cui et al. (2014)	2.7	0.5 mg/ml	Tiopronin	MDA-MB-231	225 kVp	1.04–1.44
Geng et al. (2011)	14	1.25, 2.5, 5 nM	Glucose	SK-OV-3	90 kVp 6 MV	1.44 1.3–1.37
Jain et al. (2014)	1.9	12 µM (500 µg/ml)	Thiol	MDA-MB-231	160 kVp	1.41
Jain et al. (2011)	1.9	12 µM (500 µg/mL)	Thiol	MDA-MB-231 L132 DU-145	160 kVp. 6 MV 15 MV	1.41 1.29 1.16
Joh et al. (2013)	12	1 mM	PEG	U251	150 kVp	1.3
Kaur et al. (2013)	5–9	5.5 µmol/mL	Glucose	HeLa	Gamma (60-Co) Carbon (62 MeV)	1.52 1.39
Khoshgard et al. (2014)	47–52	50 µM	PEG, Folate-conjugated	HeLa	Gamma (60-Co)	1.64 (targeted) 1.35 (untargeted)
Kong et al. (2008)	10.8	15 µM	Cysteamine/glucose	MCF-7	200 kVp 662 keV 60-Co	1.3 (cysteamine) 1.6 (glucose)
Liu et al. (2010)	6.1	0.4–1 mM	PEG	EMT-6 CT26	6.5 keV 8.048 keV 160 kVp 6 MV 3 MeV proton	2–45% decrease in survival rate
Liu et al. (2008)	4.7	500 µM	PEG	CT26	6 MV	1.33–1.59
Liu et al. (2015)	14.8	1.5–15 µg/ml	Citrate	HeLa	50 kVp X-rays 70 keV/µm carbon	1.14–2.88 1.27–1.44
Ngwa et al. (2013)	50	0.2 mg/ml	Methyl polymer	HeLa	I-125 seeds with average photon energy of 28 keV	1.7–2.3
Rahman et al. (2009)	1.9	0.25, 0.5, 1 mM	Thiol	BAEC	80 Kvp 150 kVp, 6 MV 12 MV	20 1.4 2.9 3.7
Roa et al. (2009)	10.8	15 nM	Glucose	DU-145	662 keV (137-Cs)	1.24–1.38
Taggart et al. (2014)	1.9	12 µM (500 µg/ml)	Thiol	MDA-MB-231 T98G DU-145	225 kVp	1.17–1.23 1.35–1.9 1.01–1.1
Wang et al. (2013)	13	20 nM	Glucose	A549	6 MV	1.49
Wang et al. (2015)	16 49	20 nM	Glucose	MDA-MB-231	6 MV	1.49 (16 nm) 1.86 (49 nm)
Wolfe et al. (2015)	31 × 9	0.3 OD	PEG, goserelin-conjugated nanorods	PC-3	6 MV	1.36 (targeted) 1.19 (non targeted)
Zhang et al. (2008)	30	15 nM	Glucose	DU-145	200 kVp	>1.3
Zhang et al. (2012)	4.8 12.1 27.3 46.6	0.05 mM	PEG	HeLa	662 keV (137-Cs)	1.41 (4.8 nm) 1.65 (12.1 nm) 1.58 (27.3 nm) 1.42 (46.6 nm)
Zhang et al. (2014)	<2	50 µg/ml	GSH or BSA	HeLa	662 keV (137-Cs)	1.3 (GSH) 1.21 (BSA)
In vivo						
Chang et al. (2008)	13	200 µl, 200 nM GNPs IV	Citrate	B16F10	6 MeV e^−^	Tumour growth delay
Chattopadhyay et al. (2013)	30	0.8 mg Au i.t.	PEG, HER2 Targeted	MDA-MB-361	100 kVp, 11 Gy	Tumour growth inhibition
Chen et al. (2015)	28	1.3 mg/mL i.v.	BSA	U87	160 kVp, 3 Gy @ 2 h post injection, 2 Gy @ 24 h post injection	Tumour regression
Hainfeld et al. (2004)	1.9	1.35 g Au/kg, 2.7 g Au/kg i.v.	Thiol	EMT-6	250 kVp, 26 Gy	50% and 86% long-term survival at 1.35 g and 2.7 g Au/kg
Hainfeld et al. (2010)	1.9	1.9 g/kg i.v.	Thiol	SCCVII	68 keV, 42 Gy, 157 keV, 50.6 Gy	Increase median survival (53 vs 76 days and 31 vs 49 days at 68 keV and 157 keV)
Hainfeld et al. (2013)	1.9	4 g Au/kg i.v.	Thiol	Tu-2449	100 kVp, 30 Gy	50% long-term tumour free survival
Joh et al. (2013)	12	1.25 g Au/kg i.v.	PEG	U251	175 kVp, 20 Gy	Increased median survival (28 vs 14 days)
Miladi et al. (2014)	6.6	50 µl, 50 mM i.t.	DTDTPA, DTDTPA-Gd	U87	Mean energy of 90 keV	DTDTPA increased lifespan up to 117.9% DTDTPA-Gd increased lifespan up to 473.3%
Wolfe et al. (2015)	31 x 9	100 µl, 40 µM GNPs i.v.	PEG	PC3	6 MV	Tumour growth delay
Zhang et al. (2012)	4.8 12.1 27.3 46.6	4 mg/kg i.v.	PEG	HeLa	662 keV	Tumour growth inhibition
Zhang et al. (2014)	<2	10 mg/kg i.p.	GSH or BSA	U14	662 keV	55% (GSH) and 38% (BSA) decrease in tumour volume

Comparison between GNPs’ radiosensitizing effect obtained with different energy sources, cell models and NP characteristics

### Cell cycle effects

GNPs may enhance radiosensitization by causing cell cycle disruption and inducing apoptosis. The sensitivity and consequent biological effects of radiation exposure are dependent on the cell cycle phase. Different cell cycle phases present differential radiation sensitivity with late S-phase cell being the most radioresistant and late G2 and mitosis being the most sensitive (Pawlik and Keyomarsi 2004). In response to radiation, cells activate cell cycle checkpoints in G1, S and G2 phases in order to repair genomic defects, maintaining its integrity, or prevent cell division by activating cell death mechanisms (Kastan and Bartek 2004). Materials other than gold, such as tachpyridine, have been shown to induce cell cycle arrest in G2/M phase most likely due to its metal binding activity (Turner et al. 2005). However, GNPs have been more extensively studied leading to several other reports of cell cycle distribution alterations (Geng et al. 2011; Roa et al. 2009; Kang et al. 2010; Mackey et al. 2013; Mackey and El-Sayed 2014; Ganesh Kumar et al. 2015).

So far, very few studies have been reported to analyse the effects of GNPs in the cell cycle after radiation exposure. Roa et al. (2009) found that GNPs (Glucose-GNPs, 10.8 nm) alone can promote an increase in the G2/M phase in DU-145 cancer cells. When irradiated using a Cs-137 source, G0/G1 phase has been shown to accelerate and arrest DU-145 cells in the G2/M phase. In these cells, increased expression of cyclin kinases (cyclin B1 and E) involved in the regulation of the cell cycle was found together with a decreased expression of p53 (tumour protein 53) and cyclin A.

However, most studies have been performed in the absence of radiation and showed distinct results (Wahab et al. 2014; Arnér and Holmgren 2000; Taggart et al. 2016; Kang et al. 2010; Mackey et al. 2013; Mackey and El-Sayed 2014; Ganesh Kumar et al. 2015; Cui et al. 2014). Thioglucose-coated 14-nm gold nanoparticles promoted an increase in the G2/M cell phase, compared to the control, which lead to enhanced SK-OV-3 cell sensitivity to 6 MV X-ray exposure (Geng et al. 2011). The effects of nuclear-targeted GNPs have also been investigated. The results indicated that nanoparticles alone increase in the sub-G1 population and disruption of the G1/S transition inducing apoptosis in cancer cells (Kang et al. 2010; Mackey et al. 2013). Another study involving 30-nm NLS (nuclear localization sequence)-GNPs in human oral squamous carcinoma (HSC-3) has shown an increase in S phase and a decrease in G2/M phase sub-population. In combination with 5′-fluorouracil that is active during the S phase, these cells were chemosensitized (Mackey and El-Sayed 2014). Furthermore, bacteria-mediated anti-proliferative GNPs demonstrated G2/M arrest accompanied with the inhibition of tubulin polymerization and increased activation of caspases 8, 9 and 3, in DU145 cells, suggesting increased apoptosis levels (Ganesh Kumar et al. 2015). Despite the results demonstrating GNPs’ influence in the cell cycle, there are other reports indicating no significant interference (Pan et al. 2009; Cui et al. 2014; Butterworth et al. 2010). This has been reported with 2.7-nm tiopronin-GNPs and 1.4-nm triphenyl monosulfonate-GNPs (Pan et al. 2009; Cui et al. 2014). However, the results might be cell line dependent as found using 1.9-nm GNPs (AuroVistTM) and two different cell lines, DU-145 and MDA-MB-231 cells. An increase in the sub-G1 population of DU-145 cells was seen after 48-h incubation, whereas this was not detected in MDA-MB-231 cells (Butterworth et al. 2010). Again, the coating and size of the nanoparticles induce distinct responses in the various cell lines. The variety of concentrations, coatings, materials and cell lines makes it very hard to draw any conclusions regarding the exact mechanism of action of NPs. However, the alterations induced by GNPs in cell kinetics could be associated to the accumulation in G2/M which is known to be the most radiosensitive, thus increasing GNP-mediated radiosensitization.

### DNA damage and repair

Another mechanism involved in GNP-induced radiosensitization is DNA damage and repair. Radiation itself induces double-strand breaks (DSBs) in DNA and their repair is essential for cell survival. Given the importance of DNA stability in determining cellular propagation, it is a key target for agents that attempt to halt cancer cells from dividing. Thus, cytotoxic targeting DNA agents such as cisplatin, gemcitabine and mitomycin C have been tested regarding their ability to act as radiosensitizers (Choudhury et al. 2006). The induction of DSBs has also been reported in the presence of GNP γ-H2AX foci analysis (Chithrani et al. 2010; Banáth and Olive 2003). Early DNA damage (1 h post irradiation) caused by GNPs appears to be related to its presence in the perinuclear region at the time of irradiation. However, late DNA damage (24 h post irradiation) seems to be related to other indirect processes such as radical production after interaction with water (Mcquaid et al. 2016).

Studies conducted with 50-nm citrate GNPs demonstrated increased foci number after being irradiated with 220 kVp and 6 MV energies for 4 or 24 h. The increased residual damage suggests possible inhibition or delay in DNA damage induction and/or repair which can be essential to the radiosensitization mechanism of NPs (Chithrani et al. 2010).

This has been further confirmed with 2.7-nm tiopronin-GNPs irradiated with 250 kVp X-rays. An increased residual DNA damage after being irradiated for 24 h was found in the samples where NPs were present. Contrastingly, no significant effect was observed only at 30 min post irradiation suggesting that the GNPs had no influence on the induction of DNA damage (Cui et al. 2014). Furthermore, GNPs have been found to induce DSBs in hepatocellular carcinoma cells after radiation exposure. Residual damage has also been found when the cells were irradiated in the presence of nanogold indicating influence in the repair mechanisms of the cells (Zheng et al. 2013). GNPs might not have all the same mechanisms of action and may induce distinct repair kinetics across different cell lines. For instance, BSA (bovine serum albumin)-capped GNPs induce increased γ-H2AX foci after 2 or 4 h of irradiation but no change is observed in the samples incubated for 24 h. This implies that these NPs do not influence cellular repair mechanisms (Chen et al. 2015). Other NPs such as 1.9-nm (AuroVistTM) have been reported to have no impact on the formation or repair in DSBs, neither at 1 h nor at 24 h after irradiation, in MDA-MB-231 cells (Jain et al. 2011).

Even if NPs demonstrate a radiosensitizing potential by promoting dose enhancement and potentially contributing to increased DNA DSB formation, the lack of consistency of cell lines, radiation sources and energies, treatment conditions and nanoparticles properties lead to incomparable results, making it difficult to draw a conclusion. The understanding of how different properties of the GNPs, irradiation conditions and biological response of various cell lines contribute to DNA damage and repair could further shed light on the underlying mechanism of GNP influence on DNA damage response.

### Potential impacts of bystander effects of GNP radiosensitization

In addition to direct radiation effects, communication between cells is also very important after radiation exposure. Cells that have not been directly exposed to radiation can receive signals from irradiated ones that were in the vicinity responding in a similar way to direct exposure (Najafi et al. 2014). This process is called the bystander effect and it can occur in different cell types such as endothelial cells, fibroblasts, lymphocytes and tumour cells (Havaki et al. 2015; Prise and O’Sullivan 2009; Butterworth et al. 2013). The bystander signals involved in this process may cause altered gene expression, damage in the DNA and chromosomes, cell proliferation alterations, cell death or changes in the translation process in non-irradiated cells (Najafi et al. 2014).

The main bystander signalling molecules are reactive oxygen species or nitrogen reactive species (RNS), cytokines, miRNA (micro-ribonucleic acid) or extracellular oxidized DNA (ecDNA) (Azzam et al. 2002, 2004; Barber 2011). These are released into the surrounding environment and reach the bystander cells through passive diffusion or by binding to receptors on their plasma membrane (Barber 2011; Azzam et al. 2003). Also it can occur by direct cell-to-cell contact via gap junction intercellular communication (GJIC) (Azzam et al. 2001). Furthermore, exosomes carrying miRNA are believed to mediate intercellular signalling between tumour cells and bystander cells (Melo et al. 2016; Yang et al. 2011; Umezu et al. 2013; Sánchez et al. 2015). 21 miRNAs have been found recently to be up- or downregulated after ionizing radiation exposure. Extracellular miR-1246 (micro-ribonucleic acid 1246) in particular appears to be increasing with irradiation dose. It was found to enhance proliferation and resistance in lung cancer cells by targeting death receptor 5 (DR5) although through a non-exosome associated pathway (Yuan et al. 2014). Nevertheless, soluble miRNAs involved in bystander signalling can be generated due to ROS, RNS, cytochrome c and cytokines (Hei 2015; Shao et al. 2011; He et al. 2011).

As NPs have been shown to alter cytokine and gene expression as well as ROS production, their single presence in the tumour environment could further change the way cells respond to radiation. NPs could also mediate bystander signalling, for example, small titanium dioxide NPs induce higher levels of oxidative stress and production of inflammatory cytokines. The expression of cytokine macrophage inflammatory protein-1 alpha (MIP-1α) and high-mobility group protein 1 (HMGB1) were found to be expressed in the presence of these NPs, both in vitro and in vivo (Fujiwara et al. 2015). This may play a role in enhancing oxidative stress as HMGB1 is known to be an inflammatory macrophage-secreted cytokine, which is activated by TNFα (tumour necrosis factor alpha) and IL-1β (interleukin 1 beta) (Andersson et al. 2000; Tang et al. 2009). The latter will in turn promote the secretion of HMGB1 (Tang et al. 2009). This creates a cycle that can propagate inflammation and it might also propagate bystander effects as cytokines like TNFα and IL-1β have been shown to be elevated in bystander cells (Zhou et al. 2008). The increased production of IL (interleukin) and TNFα stimulates nitrogen oxide (NO) and ROS biosynthesis by activating NF-kB (nuclear factor kappa B) transcription factor, directly or indirectly, which in turn leads to the activation of iNOS (inducible nitric oxide synthase) and COX-2 (cyclooxygenase-2) genes’ expression (Zhou et al. 2008). iNOS controls the production of NO and COX-2 is involved in ROS production, therefore increasing oxidative stress (Najafi et al. 2014; Hei et al. 2011). Moreover, small airway epithelial cells (SAECs) exposed to GNPs are able to induce protein expression in neighbouring lung fibroblasts in co-culture systems. This study found that 47 proteins were upregulated, while 62 were downregulated in the fibroblasts receiving signals from the incubated SAECs with GNPs. Most of the proteins identified are involved in cell adhesion, extracellular matrix and cytoskeleton remodelling. Plasminogen activator, PLAU (plasminogen activator urokinase), UPA (urokinase-type plasminogen activator) and GRO-1 (growth-regulated oncogene 1) that are implicated in cell migration were found to be downregulated, potentially decreasing it. Contrastingly, proteins that promote cell adhesion such as Paxillin (PXN), breast cancer anti-oestrogen resistance 1 (BCAR1) and Caveolin-1 (Cav-1) were upregulated (Ng et al. 2015).

PXN, a focal adhesion (FA), is an associated adapter protein that regulates cell spreading and motility. BCAR1 has been correlated with controlling the spread and motility of cancer cells through regulating FA (Machiyama et al. 2014; Miao et al. 2012; Schaller 2001). Furthermore, Cav-1 is known to mediate cancer metastasis (Brennan et al. 2012). Also Cav-1 can promote NF-kB activation and lung inflammatory response, through eNOS (endothelial nitric oxide synthase) and NO production (Mirza et al. 2010). These results might indicate airway inflammation since it can be related to increased adhesion molecules (Garrean et al. 2006; Liu et al. 2014). The elevated cell adhesion was accompanied by altered F-actin stress fibre arrangement in the cytoskeleton of the lung fibroblasts. Similarly, there was an increase in vinculin binding sites that are associated with F-actin anchoring to the cell membranes of the fibroblasts, which can potentially lead to enhanced vascular permeability as previously described (Snyder-Talkington et al. 2013; Pacurari et al. 2012; Setyawati et al. 2013). Overall, cytoskeleton remodelling and increased cell adhesion may affect lung function, thus reminding of the importance of understanding the cellular communication pathways in the presence of NPs even in the absence of radiation (Ng et al. 2015).

In addition to cellular bystander responses at the tissue and whole organism levels, a phenomenon called an abscopal effect can occur. There is a response of an organ/site distant from the irradiated one, where the cells are not close to each other, which has been observed in patients undergoing localized radiotherapy (Kaminski et al. 2005). The importance of the abscopal effect remains to be fully understood. It might have the potential to either increase cell killing or protect normal tissues (Prise and O’Sullivan 2009). The role of NPs in mediating abscopal effects has not been elucidated due to a lack of in vivo and clinical studies.

As nanoparticles may induce changes in cell signalling, leading to various responses depending on their size, shape and coating, understanding which signalling pathways are influenced could potentially help understand their mechanism of action in the bystander/abscopal and radiosensitization effects.

## Uptake, imaging potential and toxicity of GNPs in biological systems

### Uptake

Radiation absorption and dose deposition is thought to be partially reliant on the number of gold nanoparticles present within the cell, meaning that cellular uptake and distribution of GNPs will have a direct influence on the degree of radiosensitization observed (Chithrani and Chan 2007). This makes cellular uptake of GNPs an important metric in modifying sensitivity to radiotherapy. Modelling carried out to replicate GNP uptake equal to 1% mass in the cytosol demonstrated dose enhancement in both the nucleus and mitochondria, despite cytosol localization. It was concluded that the physical mechanism of dose enhancement was caused by photoelectron delocalization from the cytosol to cell organelles, meaning that the dose enhancement effects were not limited to the vicinity of the nanoparticles (McNamara et al. 2016).

Both uptake potential and blood circulation times have been shown to be closely associated with nanoparticle size. The optimal size for uptake has been found to be between 25 and 50 nm, with particles smaller than 10 nm or larger than 100 nm exhibiting a reduced uptake potential (Yang et al. 2014).

Chithrani et al. found the uptake of gold nanoparticles to be heavily reliant on their size and shape. Internalization of smaller particles is observed to be a more rapid process than that of larger particles. This is important as the uptake of nanoparticles into the cell will have a direct influence on the level of radiosensitization (Chithrani and Chan 2007). Coulter et al. reported the maximum amount of nanoparticle uptake to occur within the first few hours of exposure of cells to 1.9-nm GNPs, with a plateau being reached after 6 h. However, they also highlighted the difficulty in making direct comparisons with previous work as a result of the number of variables involved, as uptake may differ depending on the nanoparticle shape, size, coating, concentration and charge, as well as cell type (Coulter et al. 2012). These studies show cellular uptake to be dependent on concentration, time and cell type. Experiments carried out in hypoxic cell models saw reduced GNP uptake, thought to be linked to reduced energy production in the cells (Jain et al. 2014).

While GNPs will passively accumulate in tumours due to the EPR effect, their encapsulation within liposomes has been shown to result in higher cellular internalization (Maeda et al. 2003). Uncapped nanoparticles bind to various plasma proteins upon administration, resulting in a large number being internalized by macrophages and removed from circulation. Liposomes have a history of being used for drug encapsulation and delivery as their large size (100–200 nm) ensures that they can pack many GNPs within their lipid bilayers. Small nanoparticles encapsulated within liposomes show better passive accumulation within the tumour than non-encapsulated nanoparticles (Chithrani et al. 2010). The contents can then be released through a triggering technique, allowing the nanoparticles to penetrate the tumour tissue more effectively (Kneidl et al. 2014). Alternatively, nanoparticles capped with PEG (polyethylene glycol) alone will show an increase in their half-life in blood (Hirn et al. 2011), while HSA (human serum albumin)-conjugated gold nanoparticles have shown increased retention in the lungs and brain compared with both apoE (apolipoprotein E)-capped and citrate-stabilized nanoparticles (Schuffler et al. 2014).

In contrast to passive targeting, active targeting is the functionalization of the GNP surface with peptides, ligands or antibodies in order to preferentially target tumour cells (Schuemann et al. 2016), taking advantage of the overexpressed surface receptors of cancer cells. Not only does this increase the therapeutic ratio through achieving a greater nanoparticle concentration within the tumour, but also reduces the overall volume of gold required for treatment. Sykes et al. (2014) carried out an investigation into the impact that nanoparticle size has on active and passive targeting. Gold nanoparticles with diameters of 15, 30, 60 and 100 nm were prepared with the surface modified with either PEG or PEG conjugated to transferrin. Those nanoparticles modified with transferrin showed a significant increase in tumour accumulation in vivo, with accumulation in the 60-nm particles being 1.9 times greater than that in their passive counterparts. However, it is of note that while the PEG-only nanoparticles were slower, they also diffused deeper into the tumour. Popovtzer et al. (2016) successfully demonstrated significant improvement in tumour radiosensitivity using GNPs covalently conjugated to CTX monoclonal antibody in mice. This actively targeted the tumour with no evidence of early or delayed toxicity, confirmed by histological characterization of the tumour and adjacent tissue both 1 and 6 weeks post treatment. TNF has been covalently conjugated to gold nanoparticles, with the aim of the interaction between TNF and its receptor TNF-R1 causing active targeting of the tumour cells. Molecules of PEG-Thiol are interspersed between TNF molecules, and nanoparticles may be treated with a coating to increase cellular internalization.

Tumour blood vessels may also be promising targets, as sub-100-nm-diameter nanoparticles are expected to accumulate in the vasculature (Perrault et al. 2009). The important role of endothelial cells within the tumour vasculature makes them ideal targets with high potential clinical impact. However, a model generated by Berbeco et al. examining gold nanoparticles as tumour vascular disrupting agents found the boosted irradiation of endothelial cells alone was not viable due to the short range of the ionizing particles. The combination of tumour-targeting functionalization with image-guided radiotherapy could combat this by ensuring that the tumour has maximum levels of GNPs present during irradiation. Nanoparticle-mediated drug delivery to the tumour vasculature has been shown to have anti-metastatic effects and tumour gold content will give a good indication of overall tumour vascularity (Murphy et al. 2008). Shifting nanoparticle accumulation from the reticuloendothelial system (RES) to other organs will result in longer retention times, though unanticipated retention over prolonged periods of time may result in cytotoxic effects (Balasubramanian et al. 2010). This accumulation of GNPs may be achieved through the addition of peptides, allowing more site-specific delivery. For example, insulin has been used to improve the delivery of gold nanoparticles to target sites in the brain (Shilo et al. 2014), while other peptide-capped gold nanoparticles have been shown to pass through the blood–brain barrier using a number of mechanisms (Velasco-Aguirre et al. 2015). The ability to accurately target organ sites for uptake greatly enhances the radiosensitizing potential of GNPs.

In conclusion, while there are several benefits to passive targeting, it is noticeably less efficient in slow-growing models when compared to fast-growing models, since the former have more mature and intact blood vessels (Kunjachan et al. 2015). Cellular targeting can face problems in the way of tissue barriers which vascular targeting avoids by providing nanoparticles with direct access or binding to the overexpressed targets. Attacking the vasculature can also have the added benefit of affecting the numerous cancer cells that rely on it for growth (Kunjachan et al. 2014, 2015). GNP uptake has been shown to be reliant on several variables, including nanoparticle shape, size, charge and concentration, alongside the cell type. While the EPR effect results in the passive accumulation of GNPs within the tumour, encapsulation and specific coatings allow for increased cellular targeting and retention.

### Imaging

A consequence of the increasing use of gold nanoparticles in biomedical applications is the need for their accurate and efficient detection in both biological and tumour samples. The ability to determine nanoparticle location provides insights of uptake pathways as well as potentially identifying nanoparticle location as a cause of cytotoxicity. Accurate imaging of nanoparticle location within a sample will help achieve precise dose deposition and may also provide understanding of mechanisms behind radiosensitization. To quantify the uptake and distribution of nanoparticles within cells, several imaging techniques can be used. The physical properties of gold nanoparticles also allow them to be used as imaging agents. Miladi et al. (2014) used 2.4-nm GNPs coated with DTDTPA (dithiolated diethylenetriamine pentaacetic acid)-gadolinium chelates to combine MRI (magnetic resonance imaging) and radiosensitizing effects. The DTDTPA coating acts in preventing the clumping of GNPs along with slowing their uptake by the RES. Osteosarcoma- and gliosarcoma-bearing rats were injected with the nanoparticles, which were then monitored using MRI. The rats were then irradiated at the point when the highest content of nanoparticles was observed in the tumour. Several approaches can be used to image the uptake and distribution of GNPs throughout the cell, including localized surface plasmon resonance, photoacoustic imaging, computerized tomography, X-ray fluorescence computed tomography and electron microscopy. Each technique will have individual advantages and limitations, so it can be worthwhile to apply multiple techniques in order to improve the reliability of diagnostics and treatments (Botchway et al. 2015).

A characteristic affecting the imaging techniques that can be used with gold nanoparticles is localized surface plasmon resonance (LSPR). This is instigated by their ability to absorb and scatter specific wavelengths of light and refers to the resonance established between incident light photons and particle surface electrons. The LSPR of a nanoparticle can provide information about its overall size and structure, as when these change, so does its resonant frequency. While LSPR has a high sensitivity and relatively low cost, it can be time consuming to set up depending on the target (Petryayeva and Krull 2011). Photoacoustic imaging (PA) involves irradiating tissue using a nonionizing short-pulsed laser beam. Exogenous contrast agents absorb this energy to produce ultrasound waves, which are received using a transducer. The mechanical acoustic waves are then converted into an electronic signal which is processed to form an image. As the photoacoustic waves are only generated within the tissue sample, there is reduced background interference. PA gives higher spatial resolution and deeper imaging depth compared to fluorescence optical imaging, while the lack of ionizing radiation also makes it a safer option than computerized tomography (CT) (Wang 2008; Pan et al. 2013; Li and Chen 2015).

The strong effect demonstrated by gold nanoparticles allows them to be used as contrast agents in photoacoustic imaging. Changing the shape and size of the nanoparticles that are being used enables the tuning of the magnitude of light being absorbed and scattered and when compared with other contrast agents, such as imaging dyes and silver nanoparticles, they demonstrate a greater absorbance (Menon et al. 2013; Huang and El-Sayed 2010).

The accumulation of gold nanoparticles in tumours due to the EPR makes them well suited for photoacoustic imaging. This helps determine the location of the tumour along with assessing the vasculature and accumulation of therapeutic agents. However, the photostability of gold nanoparticles is a potential limitation as they can change shape due to high laser energies. Zhang et al. utilized GNPs as PA agents to detect human breast cancer xenografts in mice. The nanoparticles were found to accumulate within the tumours after 5 h following injection via tail vein and a significant enhancement in signal intensity was seen. This accumulation and signal enhancement would allow gold nanoparticles to be used as both tumour contrast agents and mediators of cancer therapy (Chang et al. 1999; Zhang et al. 2009).

The high atomic number and potentially long circulation time make gold nanoparticles ideal contrast agents for CT (Cormode et al. 2014). Hainfeld et al. used 1.9-nm gold nanoparticles as a contrast agent for X-ray therapy in rats. Contrast was estimated to be ~3 times greater than that of iodine at 100 keV, a useful range for clinical CT. The agent was then observed to be excreted via the kidneys with no observed toxicity. Further work used gold nanoparticles as a contrast agent to detect smaller tumours (1.5 mm). Nanoparticles were observed to accumulate in the tumours, and micro-CT allowed for quantification of this. They also noted that there was a greater uptake of nanoparticles without any attached antibody, which they believed to be due to the smaller size.

Alric et al. used a gadolinium chelate to produce a nanoparticle that could be used for both MRI and CT imaging. The nanoparticles were found to circulate freely in blood with no adverse accumulation in the lungs, liver and spleen (Alric et al. 2008).

X-ray fluorescence computed tomography (XFCT) is an imaging technique that aims to simultaneously determine the identity, quantity and spatial distribution of elements within imaged objects. Previously, Cheong et al. had been successful in accurately identifying the location of a GNP-filled object within a small animal-sized plastic phantom, as well as quantifying the amount of GNPs present. However, at the time their technique was not yet practical for routine in vivo use (Cheong et al. 2010). More recently, Manohar et al. (2016) demonstrated the use of benchtop XFCT for imaging a small animal that had been injected with gold nanoparticles. However, they found that their set-up required further refinement before it could be used routinely.

Electron microscopy is well suited to imaging gold nanoparticles due to their high electron density. It allows for the determination of the size and shape down to 1 nm and as a result is commonly used when characterizing nanoparticles. However, transmission electron microscopy (TEM) has a complicated and time-consuming sample preparation, and individual nanoparticles are not also distinguishable if their size is below the resolution limit (Schrand et al. 2010). Scanning electron microscopy (SEM) requires coating samples in a conductive metal, usually composed of nanometer-sized clusters. The similarities between samples prepared using conventional methods and metal nanoparticles make SEM incompatible. However, Goldstein et al. (2014) developed a simplified procedure that involved chromium coating, allowing them to observe cellular uptake of GNPs.

The physical properties of GNPs allow them to be used with a wide range of imaging techniques. This not only allows for the quantification of their uptake and distribution, but also makes them potential contrast agents. The advantages and limitations of each technique indicate that it may be necessary for more than one technique to be applied to improve reliability.

### Toxicity

GNPs must exhibit safe behaviour after cellular uptake to be considered potential radiosensitizers. If individual nanoparticles are found to be cytotoxic or to reduce cell viability, then they might not be suitable for clinical use. Following IV (intravenous) injection, both the biodistribution and clearance of nanoparticles are influenced by various physiological factors (bloodstream, opsonization, endothelial permeability of vessels and organs) as well as the individual physicochemical properties of nanoparticles (size, charge and surface chemistry). These physicochemical properties can be altered to control their biodistribution. It is preferable that particles are later removed through urine as this implies that there has been no degradation.

There is a level of uncertainty regarding the cytotoxicity of GNPs. While bulk gold is known to be biologically safe, functionalized GNPs have shown obvious cytotoxicity (Goodman et al. 2004). Size, concentration, cell type and treatment time are all basic parameters to be considered when examining the cytotoxicity of GNPs. Size is an important factor, as very small particles have been found to be highly toxic, and larger particles are relatively nontoxic (Pan et al. 2007).

Zhang et al. summarized that while high gold concentrations cause an obvious decrease in cell viability, low gold concentrations do not appear to influence viability. Nanoparticles with a diameter of 15 nm were found to be nontoxic up to 75 µg/ml, though cell viability was obviously affected at concentrations of gold >150 µg/ml. At 600 µg/ml, cell viability was reduced to 41.8%, compared to 93.9% at 18.75 µg/ml (Zhang et al. 2009).

A study by Connor et al. found no difference in growth rates between untreated cells and cells exposed to 25 mM concentration of 18-nm nanoparticles over the course of 5 days. The uptake and localization of the nanoparticles in the cell were confirmed by TEM, leading to the conclusion that nanoparticles are not inherently toxic to human cells. However, it was noted that the determination of whether the nanoparticles are modified by their environment is important, as this may result in significant variation to their clinical applications (Connor et al. 2005).

In 2004, Hainfeld et al. (2004) carried out work that involved injecting mice with 2.7 g Au/kg per body mass. These mice survived over a year without showing any obvious clinical effects, suggesting that in this case the nanoparticles were biocompatible. Further work by Hainfeld et al. (2013) showed that irradiation with 11.2-nm nanoparticles increased long-term survival (over 1 year) of mice by 50%, while none of the mice receiving the same treatment without nanoparticles survived longer than 150 days. As the LD50 (lethal dose at 50%) of these nanoparticles was >5 g Au/kg, a dose of 4 g Au/kg was used, showing that by using the appropriate dose of nanoparticles, toxicity can be averted and lifespan can be greatly improved.

The effects of 5- and 15-nm GNPs on mouse fibroblast cells were examined by Coradeghini et al. to provide data on the toxic potential of different sized nanoparticles. Using a colony forming efficiency (CFE) assay, the in vitro toxicity of gold nanoparticles was tested at the concentrations of 10–300 µM and at the times of 2, 24 and 72 h. Significant cytotoxicity was only seen in cells treated with 5-nm nanoparticles at a concentration over 50 µM and an exposure time of 72 h, with no significant cytotoxicity observed in the 15-nm nanoparticles. TEM imaging showed that cellular internalization occurred for both sizes of nanoparticles (Coradeghini et al. 2013).

Stefan et al. examined the effects of 12- and 22-nm chitosan-capped gold nanoparticles on rats treated with lipopolysaccharide (LPS). Brain and liver tissue reactivity was assessed following 8 days of administration. They found that while the body weight of the treated rats did not change significantly, the ratio between liver and body weights significantly increased with both nanoparticle sizes, especially the 22-nm ones, suggesting potential liver toxicity. The 22-nm nanoparticles also experienced a significant decrease in their brain-to-body weight ratio, suggesting possible brain damage. Dark-field imaging showed the agglomeration of nanoparticles within cytoplasmic cellular regions as a potential cause of this damage. This was not seen when using the 12-nm nanoparticles (Stefan et al. 2013).

While it remains a challenge to accurately estimate the cellular response to a given nanoparticle size, there are general trends which can be trusted. While both the cell type and surface properties of the nanoparticle play a role in nanoparticle uptake, smaller nanoparticles are more likely to be passively internalized, though are also more likely to have a cytotoxic effect (Shang et al. 2014).

As there is increasing interest in using nanomaterials in both research and a clinical setting it is important that nanoparticle cytotoxicity can be tested in a fast and efficient manner. Karlsson et al. used the in vivo assay “ToxTracker” with a panel of metal oxide and silver based nanoparticles. This comprises of a panel of mouse embryonic stem cells, each containing a GFP (green fluorescent protein)-tagged reporter for a distinct cellular signalling pathway. In this way it is possible to identify DNA damage caused by direct DNA interaction, oxidative stress and general cellular stress (Karlsson et al. 2014). If this assay was adapted for GNPs it may elucidate their cytotoxic properties.

## Conclusion

Despite NPs’ potential to induce radiosensitization in cancer cells, there are several challenges towards clinical translation which has, to date, led to only a few clinical trials being undertaken with the majority being liposome based and related to targeting, not involving radiosensitization (Anselmo and Mitragotri 2016). Among these challenges, there are some inconsistencies found in the mechanisms of action of different GNPs, reduced long term side effects in vivo studies and the limited demonstration of therapeutic efficacy at megavoltage energies, at which radiotherapy is clinically performed (McMahon et al. 2016).

Regardless of the distinct simulation results using megavoltage energies, an experimentally significant increase in DNA damage in the presence of GNPs has been observed. At 6 MV, the number of DSBs increases in HeLa cells, with increasing depth and as a function of the field size (Berbeco et al. 2012). Also, at MV energies it is possible to achieve dose enhancement in vivo (Chang et al. 2008; Mousavie Anijdan et al. 2013). Furthermore, a number of studies conducted at MV energies are also required prior to clinical application to support the results obtained at keV energies that most experiments are performed at. Also the dose delivered needs to be adjusted as NPs increase the dose deposition tremendously in its vicinity and in a clinical context it is usually much lower to limit exposure of organs at risk (McMahon et al. 2011). Moreover, NPs may change cellular communication influencing the clinical outcome, therefore requiring further studies in this field.

Nevertheless, using NPs can be an asset not just to radiosensitize cells but also to provide contrast as they can be imaged. This would provide a theranostic agent, combining therapeutic and imaging potential in one NP, thus improving accuracy and results of treatment delivery. Despite its potential applications, without understanding the mechanisms mediating the biological effects in cells, it is difficult to move towards clinical applications in a robust way.

## Abbreviations

AET: cysteamine
apoE: apolipoprotein E
BCAR1: breast cancer anti-oestrogen resistance 1
BSA: bovine serum albumin
Cav-1: caveolin-1
CT: computerized tomography
COX-2: cyclooxygenase-2
DSBs: double-strand breaks
DEF: dose enhancement factor
DNA: deoxyribonucleic acid
DR5: death receptor 5
DTDTPA: dithiolated diethylenetriamine pentaacetic acid
ecDNA: extracellular deoxyribonucleic acid
eNOS: endothelial nitric oxide synthase
FA: focal adhesion
GRO-1: growth-regulated oncogene 1
GJIC: gap junction intercellular communication
Glu: thioglucose
GNPs: gold nanoparticles
GFP: green fluorescent protein
GSH: glutathione
HER2: human epidermal growth factor
HSS: human serum albumin
HMGB1: high-mobility group protein 1
IL: interleukin
IV: intravenous
IL-1β: interleukin 1 beta
IMRT: intensity-modulated radiotherapy
iNOS: inducible nitric oxide synthase
LD50: lethal dose at 50%
LPS: lipopolysaccharide
LSPR: localized surface plasmon resonance
MRI: magnetic resonance imaging
miRNA: micro-ribonucleic acid
miR-1246: micro-ribonucleic acid 1246
MIP-1α: macrophage inflammatory protein-1 alpha
NF-kB: nuclear factor kappa B
NO: nitrogen oxide
NPs: nanoparticles
PA: photoacoustic imaging
PDI: protein disulfide isomerase
PEG: polyethylene glycol
PLAU: plasminogen activator urokinase
PNX: paxillin
p53: tumour protein 53
RES: reticuloendothelial system
ROS: reactive oxygen species
RNS: reactive nitrogen species
SAECs: small airway epithelial cells
SEM: scanning electron microscopy
TEM: transmission electron microscopy
TrxR1: thioredoxin reductase 1
TNFα: tumour necrosis factor alpha
UPA: urokinase-type plasminogen activator
XFCT: x-ray fluorescence computed tomography
